# Contrasting Patterns of Clinal Genetic Diversity and Potential Colonization Pathways in Two Species of Western Atlantic Fiddler Crabs

**DOI:** 10.1371/journal.pone.0166518

**Published:** 2016-11-18

**Authors:** Claudia Laurenzano, Tânia M. Costa, Christoph D. Schubart

**Affiliations:** 1 Zoology, University of Regensburg, Regensburg, Germany; 2 Department of Biology, University of Louisiana at Lafayette, Lafayette, Louisiana, United States of America; 3 Biosciences Institute, São Paulo State University (UNESP), São Vicente, São Paulo, Brazil; National Cheng Kung University, TAIWAN

## Abstract

Fiddler crabs (Brachyura, Ocypodidae), like many other marine organisms, disperse via planktonic larvae. A lengthy pelagic larval duration is generally assumed to result in genetic connectivity even among distant populations. However, major river outflows, such as of the Amazon or Orinoco, or strong currents may act as phylogeographic barriers to ongoing gene flow. For example, the Mona Passage, located between Puerto Rico and Hispaniola, has been postulated to impair larval exchange of several species. In this study, Cox1 mtDNA data was used to analyze population genetic structure of two fiddler crab species from the western Atlantic, comparing the continental coastline and Caribbean islands. The results indicate genetic homogeneity in *Minuca rapax* among Atlantic (continental) populations (Suriname, Brazil), whereas Caribbean populations show significantly restricted gene flow among the constituent islands and towards continental populations. Our data support the hypothesis of the Mona Passage hindering larval exchange. Contrastingly, Caribbean *Leptuca leptodactyla* populations appear to be devoid of detectable variation, while Atlantic-continental (i.e. Brazilian) populations show much higher haplotype and nucleotide diversities and display slight genetic differentiation among populations within the Atlantic region, though not statistically significant. Both species show a pronounced divergence between regions, supporting the presence of a phylogeographic barrier.

## Introduction

Ongoing gene flow among widespread populations is essential for genetic homogeneity within a species, while its disruption leads to genetic differentiation and heterogeneity. Being rather limited in spatial expansion as adults, a dispersive planktonic larval stage is part of the reproductive strategy of many coastal marine organisms [1–3]. The longer the timespan that larvae spend in the plankton, the greater the distances they can be transported by water currents, and thus, the larger the geographic range within which gene flow counteracts genetic structuring [4, 5]. The hypothesis that the pelagic larval duration (PLD) and the genetic structure stand in a close-knit correlation was already discussed decades ago [6–9]. However, a number of studies has shown that a lengthy PLD does not necessarily guarantee high levels of population connectivity [10–14]. In a recent review [15], Weersing and Toonen pointed out that there is indeed an undeniable link in this matter, but that there are numerous other factors that bias the degree of larval exchange and therefore the scale of genetic differentiation.

In marine ecosystems, barriers to dispersal are much less obvious compared to terrestrial ones. Oceanographic features such as major currents, ocean fronts, or eddies can severely impair larval migration [10, 16, 17]. Taylor and Hellberg [18] suggest these characteristics to hold responsibility for evident gene flow restriction across the Mona Passage between Hispaniola and Puerto Rico. This area has been reported to genetically divide the Caribbean realm into west and east by multiple studies (e.g. [12, 19, 20]). Immense freshwater outfluxes of major rivers such as the Amazon or Orinoco may jeopardize larval development or survival through altered salinity or temperature levels, or by washing propagules far offshore [10, 21]. A number of fish genera are found to have endemic sister species in Brazil and the Caribbean, respectively, proposing the Amazon to impede ongoing gene flow between the areas (see [22] and citations therein). Similarly, decapods have been shown to exhibit genetic structuring between these two regions (e.g. caridian shrimp [23]).

Fiddler crabs (Brachyura, Ocypodidae, formerly known as genus *Uca*, now represented in several genera, [24]) release their young during nocturnal spring high tides of large amplitude [25–27], presumably preventing larval retention within the estuary [28]. Larvae are washed offshore and spend several weeks in the plankton carried by surface ocean currents [1, 29, 30], before they undergo metamorphosis to the first crab stage in suitable habitats [31–34]. Recent studies on population structuring in fiddler crabs disclosed high genetic connectivity within large ranges. [35] report lack of differentiation in *Austruca occidentalis*, formerly known as *U. annulipes*, along an East African latitudinal gradient of 3,300 km. A comparison between Brazilian and Argentinean populations of *Leptuca uruguayensis* (see [36]) showed genetic homogeneity despite a distance of 2,000 km and the discharge of the Río de la Plata, a postulated biogeographic barrier to various decapods [37, 38]. Research on other fiddler crabs along the Brazilian coast was unable to detect structuring among examined communities. However, while no significant genetic variance could be detected along the entire coastal range of Brazil, significant morphometric differences were evident for almost all local fiddler crab species [39, 40].

The distribution of the mudflat fiddler crab *Minuca rapax* ranges from the Gulf of Mexico, south Florida and the West Indies to Santa Catarina, Brazil [41–45], where they colonize softer sediments such as mud or clayey areas in mangroves or marshland [41, 45]. A previous study on its genetic structure [46] revealed genetic homogeneity within Suriname and Brazil. Yet, Caribbean island populations appeared to differ significantly from each other as well as from the above mentioned mainland populations. Nonetheless, the question if the Mona Passage constitutes a barrier to the species’ dispersal within the Caribbeanhas not been addressed.

The thin-fingered fiddler crab *Leptuca leptodactyla* occurs from the Caribbean along the coast of Venezuela as far south as Santa Catarina State (Brazil). Contrasting to *M. rapax*, this species inhabits larger-grained substrate like sand [41, 45] and is often found in areas without vegetation coverage (*pers. obs.*). The two fiddler crab species share a similar life history [47] (planktonic larval development of two to three weeks, [48], S. Brandt Martins, U.G. Silva & S. Masunari unpublished data) as well as overlap in range for large parts of their respective spatial distribution—thus, similar patterns of genetic structure or connectivity may be expected.

This study addresses the matter of *a priori* postulated biogeographic barriers within the tropical western Atlantic, such as the Amazon and Orinoco rivers and their impact on the population structure of two fiddler crab species that share similar reproductive strategies and a large part of their geographic distribution, yet show slight differences in their respective ecological preferences. With an increased dataset compared to [46], i.e. among others a population from Puerto Rico, this study also aims to further elucidate the genetic connectivity of *M. rapax* in the Caribbean and tests the Mona Passage as a possible impediment on gene flow between western (Dominican Republic) and eastern (Puerto Rico) populations. Despite the capability for large-scale genetic connectivity due to a lengthy PLD, populations of both species, respectively, on opposite sides of such potential barriers are expected to show evidence of gene flow restrictions when compared with one another. Furthermore, since previous results indicated interrupted genetic exchange in Caribbean populations of *M. rapax*, similar results can be expected for *L. leptodactyla*.

## Materials and Methods

### Sampling and Molecular Methods

**Fig 1:**
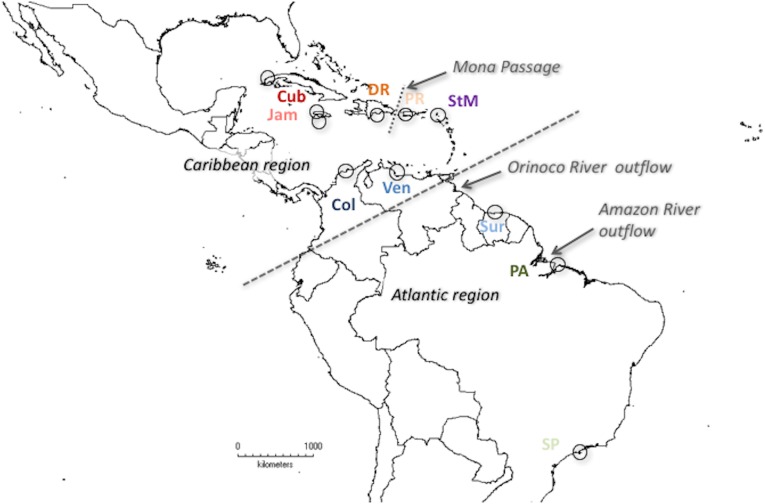
Sample sites of *Minuca rapax* *Uca rapax*, represented by circles. Cub—Cuba, Col—Colombia, DR—Dominican Republic, Jam—Jamaica, PA—Brazil (Pará State), PR—Puerto Rico, SP—Brazil (São Paulo State), StM—St. Martin, Sur -Suriname, Ven—Venezuela. Arrows point to potential biogeographic barriers, dashed line indicates suggested geographical regions.

*Minuca rapax* specimens were obtained from Cuba, Jamaica, the Dominican Republic, Venezuela, and the Brazilian federal states Pará and São Paulo. For a more complete analysis, samples from St. Martin and Suriname were obtained as a loan from the Naturalis Museum Leiden (RMNH-D 32206 and 12415, respectively), the specimen from Colombia was loaned to us by the Senckenberg Museum Frankfurt (SMF 6864). New to the current study (compared to [46]) are specimens from Puerto Rico, which allow us to compare populations from opposite sides of the Mona Passage and, thus, draw conculsions on the role of said passage as potential barrier to dispersal. In most cases, at least ten representatives of each population were included, except for the Cuban population, for which only nine individuals could be PCR-amplified(Fig 1).

**Fig 2:**
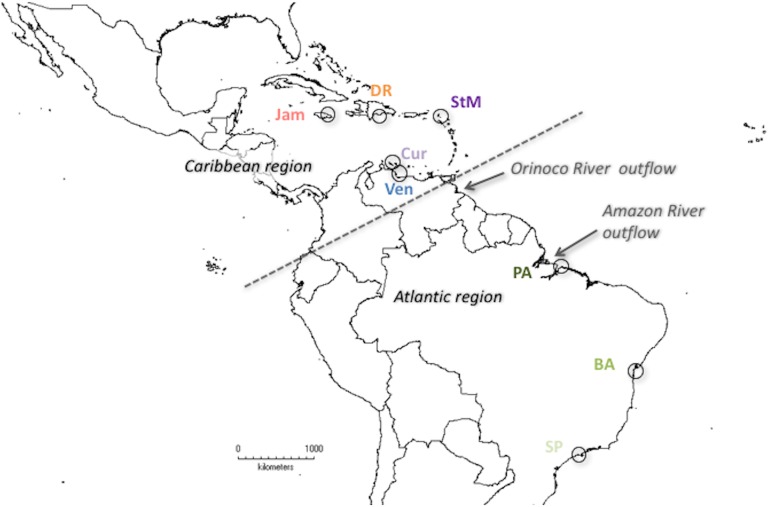
Sample sites of *Leptuca leptodactyla*, represented by circles. BA—Brazil (Bahia State), Cur—Curaçao, DR—Dominican Republic, Jam—Jamaica, PA—Brazil (Pará State), SP—Brazil (São Paulo State), StM—St. Martin, Ven—Venezuela. Arrows point to potential biogeographic barriers, dashed line indicates suggested geographical regions.

Specimens of *Leptuca leptodactyla* used in this study are from Jamaica, the Dominican Republic, Venezuela, and the Brazilian federal states Pará, Bahia, and São Paulo. Additional animals from St. Martin and Curaçao were added through museum loans from the Naturalis Museum Leiden (RMNH-D 12739 and 1001, respectively) (see Fig 2). Both studied species are common coastal organisms, with marine larval development and wide distribution. Thus, they are not endangered or protected in any of the collected countries. Furthermore, im most cases, only single pereiopods were removed for genetic analyses and the animals released.

Genomic DNA was extracted from muscle tissue of pereiopods using the Purgene method (Gentra Systems). An 897 basepair (bp) region encoding the 3’end of *cytochrome c oxidase subunit 1* was amplified for nearly all samples by means of polymerase chain reaction (PCR) (40 cycles; 45 sec 94°C, 1 min 48°C, 75 sec 72°C denaturing, annealing, elongation temperatures) with the primers COL1b 5’-CCW GCT GGD GGW GGD GAY CC-3’and COH16 5’-CAT YWT TCT GCC ATT TTA GA-3’[49]. Of one sample however (R 836-21, Colombia), only a shorter fragment (650 bp) of the same gene could be obtained by using the primer combination COL1b and COH1b 5’-TGT ATA RGC TRC TGG RTA RTC-3’[49]. PCR products were outsourced for purification and sequencing to LGC, Eurofins, GATC, or Macrogen, using dideoxy chain termination sequencing with primer COL1b.

### Population Genetic Analyses

**Fig 3:**
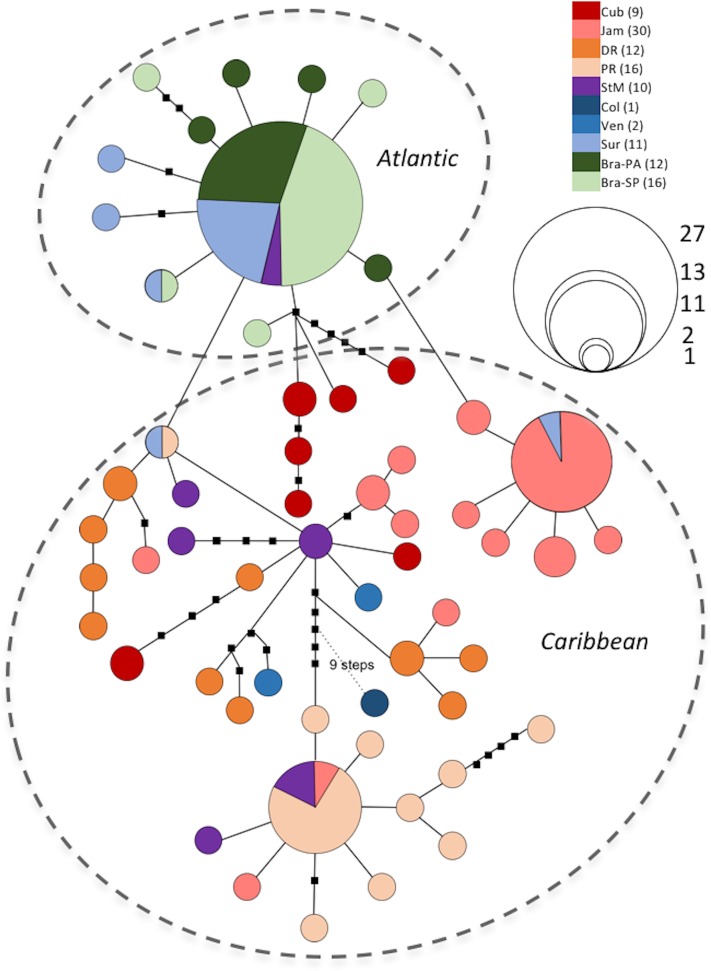
Haplotype network of *Minuca rapax* constructed with TCS 1.21. Derived from CO1 mtDNA (825 bp, N = 119), 95% connection limit. Solid-lined circles represent sampled haplotpyes according to frequency and populations: Bra-PA—Brazil (Pará State), Bra-SP—Brazil (São Paulo State), Cub—Cuba, Col—Colombia, DR—Dominican Republic, Jam—Jamaica, PR—Puerto Rico, StM—St. Martin, Sur -Suriname, Ven—Venezuela. Reticulations were dissolved in favor of shortest distances. Dashed circles suggest geographic regions.

**Fig 4:**
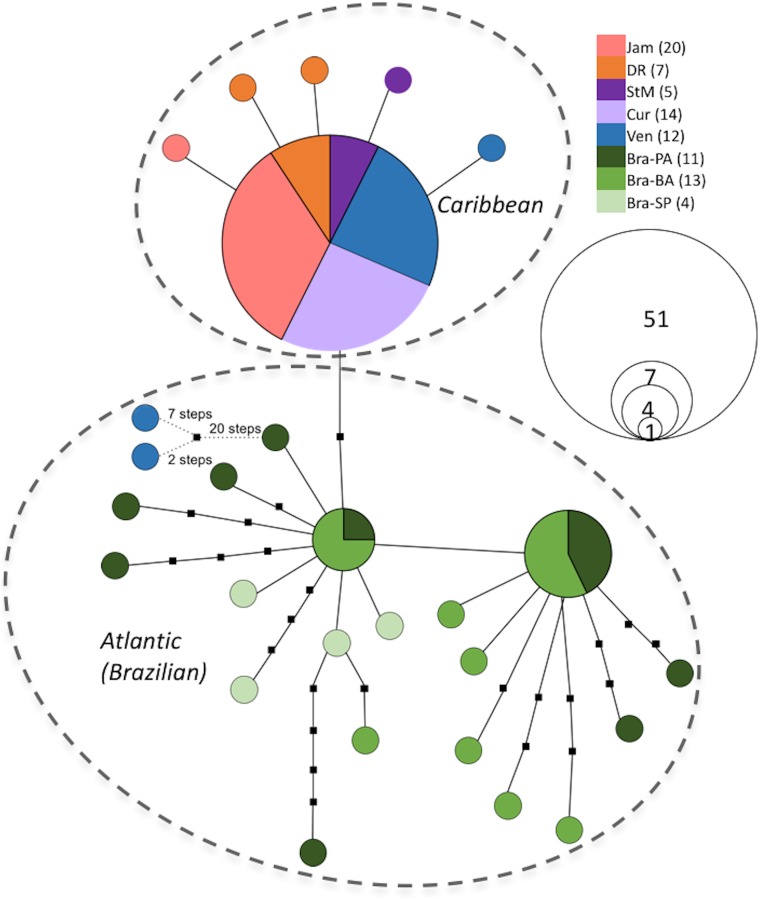
Haplotype network of *Leptuca leptodactyla* constructed with TCS 1.21. Derived from CO1 mtDNA (825 bp, N = 86), 95% connection limit. Solid-lined circles represent sampled haplotpyes according to frequency and populations: Bra-BA—Brazil (Bahia State), Bra-PA—Brazil (Pará State), Bra-SP—Brazil (São Paulo State), Cur—Curaçao, DR—Dominican Republic, Jam—Jamaica, StM—St. Martin, Ven—Venezuela. Dashed circles suggest geographic regions.

Obtained DNA sequences were proofread with Chromas Lite 3.01 (Technelysium Pty Ltd., 2005) and then aligned with BioEdit 7.0.9.0. [50]. Non-readable parts in the beginning and primer regions were omitted. The resulting dataset for *M. rapax* contains 119 sequences of 825 bp for the primer combination COL1b/COH16 and one sequence of 608 bp for COL1b/COH1b, while the dataset for *L. leptodactyla* comprises 90 sequences of 825 bp. The absence of stop codons, which might indicate the presence of pseudogenes, was checked using the software Artemis [51]. Sequences of each haplotype were submitted to EMBL Nucleotide Sequence Database. *M. rapax* sequences are published under the accession numbers LM651222 to LM651237 as well as HE972299 to HE972339; *L. leptodactyla* sequence accession numbers are LN610512 to LN610538. For population genetic analyses, CO1 data of 825 bp length were used to construct a haplotype network with TCS 1.21 [52] and to apply an analysis of molecular variance (AMOVA) using Arlequin 3.5.2.1. [53]. Four *M. rapax* sequences (R 584-9, Suriname; R 804-7 and 14, Venezuela; R 836-21, Colombia) and four *L. leptodactyla* sequences (R 411-1, 2, R 763-7, Bahia; R 756-3, Pará) could not be included in the AMOVA either because of poor quality of the sequence (less than 770 bp readable) or because of insufficient sample size of their population. The same datasets were used to assess haplotype and nucleotide diversities of the respective populations with DnaSP 5.10.1. All parameters are shown in the table head (Tables 1–5) and the figure captions (Figs 3 and 4), respectively.

**Table 1:** Estimate of pairwise differences among *Minuca rapax*, derived from CO1 mtDNA (825 bp, N = 115). P values above diagonal, *ϕ*_*ST*_ values below diagonal. Significance level 0.05; +++: p < 0.001, +: p <0.05, -: not significant. Significant *ϕ*_*ST*_ values in bold type. Numbers in parentheses according to sample size. Arlequin 3.5.1.3. Groups define geographic regions as follows: Caribbean (Jam, Cub, DR, PR, StM) vs. Atlantic (Sur, Bra). Abbreviations as in Fig 1, Bra—Brazil (pooled data from PA, SP).

	Jam (30)	Cub (9)	DR (12)	PR (16)	StM (10)	Sur (10)	Bra (28)
Jam		+++	+++	+++	+++	+++	+++
Cub	**0.368**		+++	+++	+++	+++	+++
DR	**0.28**	**0.258**		+++	+++	+++	+++
PR	**0.491**	**0.484**	**0.438**		+	+++	+++
StM	**0.274**	**0.244**	**0.184**	**0.1**		+++	+++
Sur	**0.522**	**0.3**	**0.399**	**0.65**	**0.417**		-
Bra	**0.586**	**0.419**	**0.532**	**0.722**	**0.548**	0.01	

Percentage of variation a) among regions: 24.09, b) among populations within regions: 26.99, c) within populations: 48.92. Fixation indices: FSC: 0.356, FST: 0.511, FCT: 0.241.

**Table 2:** Haplotype and nucleotide diversity of *Minuca rapax* calculated with DnaSP ver. 5.00.07. 5,000 replicates. Abbreviations as in Fig 1.

	N	h	hd	P_*i*_
**Jam**	30	13	0.821	0.003
**Cub**	9	7	0.944	0.006
**DR**	12	12	0.97	0.005
**PR**	16	9	0.767	0.003
**StM**	10	8	0.956	0.004
**Sur**	10	5	0.576	0.001
**PA**	12	5	0.576	0.001
**SP**	16	5	0.45	0.001
**Σ**	115	54	0.925	0.005

**Table 3:** Estimate of pairwise differences with Arlequin 3.5.1.3 among Caribbean populations of *Leptuca leptodactyla*, derived from CO1 mtDNA (825 bp, N = 58). P values above diagonal, *ϕ*_*ST*_ values below diagonal. Significance level 0.05; -: not significant. Numbers in parentheses correspond to sample size. Abbreviations as in Fig 2.

	Jam (20)	DR + StM (12)	Cur (14)	Ven (12)
Jam		-	-	-
DR + StM	0.027		-	-
Cur	- 0.019	0.013		-
Ven	0.133	0.071	0.092	

**Table 4:** Estimate of pairwise differences with Arlequin 3.5.1.3 among pooled Caribbean (see Table 3) and Atlantic populations of *Leptuca leptodactyla*, derived from CO1 mtDNA (825 bp, N = 82). P values above diagonal, *ϕ*_*ST*_ values below diagonal. Significance level 0.05; +++: p < 0.001, -: not significant. Significant *ϕ*_*ST*_ values in bold type. Numbers in parentheses correspond to sample size. Groups define geographic regions as follows: Caribbean (Jam, DR + StM, Cur, Ven) vs. Atlantic (BA, PA). Abbreviations as in Fig 2.

	Car (58)	PA (11)	BA (13)
Car		+++	+++
PA	**0.465**		-
BA	**0.517**	0.004	

Percentage of variation a) among regions: 45.14, b) among populations within regions: 2.18, c) within populations: 52.68. Fixation indices: FSC: 0.4, FST: 0.473, FCT: 0.451.

**Table 5:** Haplotype and nucleotide diversity of *Leptuca leptodactyla* calculated with DnaSP ver. 5.00.07. 5,000 replicates. Abbreviations as in Fig 2.

	N	h	hd	P_*i*_
**Jam**	20	2	0.1	<0.001
**DR**	7	3	0.524	0.001
**StM**	5	2	0.4	<0.001
**Cur**	14	1	<0.001	<0.001
**Ven**	10	2	0.2	<0.001
**PA**	11	9	0.945	0.005
**BA**	13	8	0.885	0.003
**Σ**	80	21	0.588	0.003

## Results

The graphical representation of the two species’haplotypes (Figs 3 and 4 are on the one hand similar in that they both show a distinct separation between the two respective geographic regions, i.e. the Caribbean region and the Atlantic region (see captions of Tables 1 and 4, respectively, for details on geographic regions). On the other hand, the two haplotype networks are strikingly contrasting when one takes a closer look at respective haplotype distributions.

### *Minuca* *rapax*

As for *Minuca rapax* (Fig 3), Atlantic specimens are all in very close mutual genetic proximity, while Caribbean haplotypes are more widespread around the network. The most common haplotype is shared by 27 individuals, all but one (St. Martin) from Atlantic populations. All remaining Atlantic haplotypes radiate from this haplotype in one- to four-step distances, forming a star-like shape with a maximum distance of six mutational steps to each other.

Caribbean haplotypes show a more disjunct distribution than the ones from the Atlantic. Maximum distances between haplotypes of the same population range from nine (Dominican Republic) to 20 (Cuba) mutational steps. Two clusters stand out: One haplotype is shared by mostly Jamaican and one Surinamese specimen, from which several Jamaican haplotypes radiate in a one-step distance. The other conspicuous cluster shows a common haplotpye present in mostly Puerto Rican individuals, as well as in some from St. Martin and Jamaica. All but one Puerto Rican haplotype are in very close genetic proximity to it. Exclusively specimens from St. Martin are found sharing haplotypes with both Atlantic and Caribbean populations, thus displaying no clustering. Different from all other populations, Cuban and Dominican haplotypes are very concentrated, i.e. haplotypes are not shared with other populations.

This pattern is reflected in the haplotype and nucleotide diversities of the respective populations (Table 2) and in the analyses of molecular variance (AMOVA) (Table 1). Diversity values are lowest for Atlantic populations and much higher for Caribbean populations. Total diversities for the *M. rapax* dataset are rather high (hd = 0.925, P_*i*_ = 0.005), confirming the depicted structure of haplotypes (see Table 2). An overall lack of differentiation among Atlantic populations becomes apparent, while Caribbean populations depict significant levels of differentiation when compared among the region, and when compared to Atlantic populations (see Table 1).

Brazilian populations (Pará and São Paulo) of *M. rapax* were pooled as they showed no significant difference towards each other in a preliminary AMOVA (*ϕ*_*ST*_ = −0.007, p > 0.05, not displayed here). In addition, two thirds of the Pará population and three quarters of the São Paulo population, respectively, share the same (main) haplotype as shown in the parsimony network. Table 1 shows significant differentiation among all populations, the comparison of the two Atlantic populations Brazil and Suriname being the only exception (p > 0.05). When comparing Puerto Rico with the other populations the difference is lowest between Puerto Rico and St. Martin, and highest between Puerto Rico and Brazil (*ϕ*_*ST*_ = 0.722). Within the Caribbean populations, Puerto Rico shows the highest divergence in pairwise comparison to populations to the west. In summary, genetic divergence is most pronounced between Atlantic and Caribbean populations. Within Atlantic populations, along the coast from southern Brazil to Suriname, continuous gene flow seems to be maintained, while Caribbean populations show significant differences in pairwise comparisons.

### *Leptuca* *leptodactyla*

The parsimony network of *L. leptodactyla* (Fig 4) shows a division of haplotypes into three distinct clusters, i.e. one Caribbean cluster and two Atlantic(Brazilian) ones. All three clusters are found in immediate genetic vicinity being one and two mutational steps apart from each other. The Caribbean cluster consists of the most common haplotype which is carried by 51 specimens of exclusively Caribbean origin (Jamaica, the Dominican Republic, St. Martin, Curaçao, and Venezuela) with five singleton haplotypes in a one-step distance. The first Brazilian cluster contains one haplotype shared by four Brazilian individuals from which multiple single-individual haplotypes radiate in distances ranging from one to six steps. Interestingly, two Venezuelan haplotypes are connected to this cluster by a chain of more than 20 missing haplotypes. The second Brazilian cluster is located one step from the four-individual haplotype. Its main haplotype is carried by seven individuals. From it, seven singleton haplotypes exclusively from Pará and Bahia, radiate in one- to three-step distances. The population from São Paulo seems to be the only one not sharing haplotypes with other populations, nor repeating haplotypes.

The structure of the haplotype network is reflected in the computed haplotype and nucleotide diversities. While diversity values are very low for Caribbean populations, they are strikingly high for Atlantic populations. Overall diversities for *L. leptodactyla* are moderate (hd = 0.588, P_*i*_ = 0.003; Table 5). Similar to *M. rapax*, AMOVA results for *L. leptodactyla* (Table 4) show a distinct separation between Atlantic and Caribbean populations. Another similarity of both species’AMOVA is the overall lack of differentiation among Atlantic populations. However, while Caribbean populations of *M. rapax* seem to be rather diverse, *L. leptodactyla* populations from the same area appear genuinely homogeneous (Table 3; variation among populations within region = 2.18%, Table 4).

Caribbean *L. leptodactyla* did not exhibit significant differentiation in pairwise comparison (*ϕ*_*ST*_ < 0.014, p > 0.05, see Table 3; individuals from the Dominican Republic and St. Martin were pooled due to their small sample size). In contrast, significant divergence could be detected between both Brazilian and the pooled Caribbean populations (*ϕ*_*ST*_ approx. 0.5, p < 0.001), while pairwise comparisons between Pará and Bahia proved to be insignificant (p > 0.05) (Table 4). Much alike *M. rapax*, these findings match the results from the parsimony network obtained.

## Discussion

### Atlantic region: the Amazon

This study compares three Atlantic populations from three Atlantic mainland sites (Suriname, Brazilian states Pará and São Paulo) and seven Caribbean populations (insular as well as continental) of *Minuca rapax*, and *Leptuca leptodactyla* populations from five Caribbean and three Atlantic(Brazilian) localities. As expected from previous studies on the genetic structure of multiple fiddler crab species in this area [36, 39, 46], *M. rapax* exhibits a clear lack of genetic structuring along the Atlantic mainland coast. Two thirds of Surinamese and Brazilian specimens share the same haplotype, the remaining third differs in very few positions (Fig 3). Pairwise comparisons corroborate this pattern, Suriname and Brazilian populations prove not to be significantly different from each other (Table 1).

Apparently, gene flow within this region is unimpaired for *M. rapax*, despite possible derogating effects of the Amazon river or local ocean currents: At Ponta do Calcanhar, Rio Grande do Norte (Brazil), the Central South Equatorial Current (CSEC) splits into the northwestward-flowing North Brazil Current and the South Brazil Current running southwestward [54]. Wieman et al. [39] addressed the population structure of the Brazilian fiddler crab (*Uca maracoani*) along the Brazilian coast with respect to the CSEC system as a potential barrier and found a lack of genetic differentiation among the compared populations. Their resulting haploype network shows a very similar structure to the findings on *M. rapax* in this study. Brazilian haplotypes of both species (and Surinamese ones in the case of *M. rapax*) form a star-like pattern, suggesting recent expansion emanating from one or few founder populations [55].

Compared to the results on *M. rapax*, the Atlantic populations of *L. leptodactyla* appear slightly less homogeneous. Haplotype diversities are much higher for Atlantic *L. leptodactyla* populations (Pará 0.945, Bahia 0.885; Table 5) than values of *M. rapax* populations from the same region (0.45 to 0.576, see Table 2). This is reflected in the haplotype network (Fig 4). The two haplotypes shared by several Brazilian individuals from which multiple rare haplotypes divert, combined with no significant gene flow restriction between Pará and Bahia, may indicate a bottleneck event in the species’history that was followed by expansion from two ancestral, divergent lineages (see [56]). It should be noted that the Sáo Paulo population may turn out to be distinct from its northward neighbors, because the sampled four individuals display unique haplotypes and are currently not represented in the common haplotypes with specimens from other Brazilian localities. Unfortunately, the population’s small sample size precludes further statistical analyses, permitting merely the anticipation of divergence.

Other Brazilian fiddler crabs sampled along this or a similar gradient (see aforementioned references), including *M. rapax*, showed genetic homogeneity. From the results obtained in this study, it cannot be inferred at this point that the Amazon river functions as a barrier to gene flow in the two studied fiddler crabs. Yet, although Wieman et al. [39] did not find evidence for significant genetic distinction, theyas well as Hampton et al [40]. detected significant morphometric differentiation that may hint at speciation commencement or phenotypic plasticity as response to ecological parameters [57]. Nevertheless, genetic differentiation has been reported for other crustaceans in this area (penaeid shrimp, see [58]; *Neohelice granulata*, see [59]).

### Caribbean region: the Mona Passage

During the past three decades, many studies postulated unhindered gene flow and consequent high levels of population connection within the Caribbean [13, 60–62]. Concordant with these findings,*L. leptodactyla* shows extremely low variability among Caribbean populations, as depicted in the haplotype network (Fig 4) and corresponding haplotype diversities (Table 5). Of all specimens sampled at Caribbean sites (Jamaica, Dominican Republic, St. Martin, Curaçao, and Venezuela), 91% share the same haplotype which is simultaneously the most common haplotype found in all tested individuals. Five rare Caribbean haplotypes radiate from it in one-step distances, forming a star-burst shape. Levels of genetic differentiation, estimated in an AMOVA (Table 3), confirm this pattern with insignificant (*p* > 0.05) estimates among all Caribbean populations, indicating ongoing gene flow. Results suggest the here studied Caribbean islands as well as Venezuela to be genetically well connected, therewith challenging Briggs [63] view of the West Indies Province (including most of the islands) being biogeographically distinct from the Caribbean Province (Central American and northern South American mainland coast, along with offshore islands from the continental shelf). This is not surprising due to the extended pelagic larval stage of the crabs’early life history. Previous studies on other species report the same outcome for similar sites within the region [13, 61, 62]. [62] and [64] even found populations within opposing current tracks to be genetically undifferentiated, proposing that contemporary major currents either do not obstruct larval exchange, or have not acted long enough to show an effect. The star-like haplotype structure suggests a bottleneck incident or rapid expansion from one or few founder communities in the history of Caribbean *L. leptodactyla* (see [4]).

Results from within the Caribbean could not be more contrasting between *L. leptodactyla* and *M. rapax*. While the former shows genetic homogeneity in the Caribbean Sea, differentiation among *M. rapax* from the same region becomes evident, their haplotypes being mostly rare. These findings are reflected in the estimated haplotype diversities (Table 2), showing the lowest values for Atlantic populations, whereas diversity in Caribbean haplotypes is much higher. Of these, Puerto Rico and Jamaica are least diverse, while Cuba and the Dominican Republic have higher estimates. The populations of Cuba and the Dominican Republic both have endemic haplotypes, which may hint at closed populations that have been self-sustained for a long evolutionary time. This may be due to localized recruitment or coastal gyres that preclude larvae from emigration (see [58]) for work on Brazilian shrimps). Gene flow within the Caribbean realm seems to be either ongoing or a legacy of the past, but significantly restricted in this fiddler crab. Recent studies revealed similar results on population structure of other marine organisms [12, 14, 18, 65–67].

Evidence from these publications and results from the current study on *M. rapax* contest the notion of the Caribbean region as a genetically uniform zone without obvious biogeographic barriers to dispersal. Instead, evidence is mounting in favor of one *a priori* postulated barrier in particular: The Mona Passage, located between Hispaniola and Puerto Rico, has been proposed to affect various marine taxa dispersing via pelagic larvae. In his work on neon gobies, [19] already observed discontinuities in color forms of *Elacatinus evelynae*. During further studies, the perception of a division between western and eastern Caribbean began to emerge [20, 68, 69]. Recent molecular research demonstrates discontinued gene flow, suggesting impaired larval interchange along the Mona Passage [12, 14, 18, 66, 70].

In the present study, the nature of Mona Passage as a barrier to gene flow in *M. rapax* is investigated with an increased data set compared to previous research [46]. Differences in pairwise comparison of Puerto Rico with western and eastern Caribbean populations elucidate the matter in question. While the degree of differentiation is significant, but very low between Puerto Rico and the Lesser Antillean island St. Martin (*ϕ*_*ST*_ = 0.1, see Table 1), differences are strikingly greater when compared to other Greater Antillean populations (*ϕ*_*ST*_ = 0.438, Dominican Republic; *ϕ*_*ST*_ = 0.484, Cuba; *ϕ*_*ST*_ = 0.491, Jamaica). Especially the difference to the Hispaniolan population is remarkable, as the two islands are close-by neighbors. [12] even detected genetic separation in *E. evelynae* from Puerto Rico and Isla Desecheo, an island only 23 km off the Puerto Rican coast, herewith presenting first molecular endorsement for a barrier in this particular region. The means by which Mona Passage functions as a break remains uncertain, the authors suggest “strong currents” coupled with “complex eddies” to bar larvae from transgressing ([18], p. 703). While we found strong evidence in favor of the Mona Passage impeding gene flow between western and eastern *M. rapax* populations, it remains untested whether the passage has a similar effect on *L. leptodactyla* due to a lack of appropriate samples available to our research at the time this study took place. This matter should be resolved in future studies.

The entirely different patterns of Caribbean populations of these two fiddler crab species are striking. While *L. leptodactyla* seems vastly homogeneous, populations of *M. rapax* appear to be very heterogeneous. The former species prefers sandy sediments closer to the waterline, the latter species, on the other hand, is found in muddier areas in mangroves and marshland [41, 45]. As previously mentioned, the availability of suitable adult habitat may be a determining factor in colonizing land and, thus, ongoing exchange among populations. The said availability may simply differ for the two species. Another possible explanation for the dissimilar population structure may lie in the paleohistory of the area. During Pleistocene glaciations, *M. rapax* and *L. leptodactyla* could have drawn back to different refugia and consequently exhibit different re-colonization patterns. The higher level of structuring in the Caribbean region combined with a rather homogeneous pattern in the Atlantic region, *M. rapax* may have survived the ice age in the Caribbean and from there re-colonized the Atlantic region. The pattern is reversed for *L. leptodactyla*, hinting at a glacial refugium in the Atlantic region and re-colonization in the Caribbean.

### Atlantic vs. Caribbean: the Orinoco

While the two fiddler crab species show contrasting results within regions, they both show a pronounced genetic divergence between regions, i.e. the Caribbean vs. Atlantic. With very few exceptions, haplotypes are not shared between regions in *M. rapax* (Fig 3), and not at all in *L. leptodactyla* (Fig 4), resulting in highly significant *ϕ*_*ST*_ values that indicate restricted gene flow of moderate to high degrees between the two groups (Tables 1 and 4). Unfortunately, only two specimens of *M. rapax* from Venezuela and one from Colombia were obtained, foreclosing further statistical analyses with these populations. Nonetheless, the rare haplotypes of these individuals rather group with other Caribbean rare haplotpyes than with Atlantic ones, indicating a trend towards a genetic division somewhere between Venezuela and Suriname. Notwithstanding the very small sample size of Venezuelan and Colombian individuals, respectively, the genetic remoteness of their haplotypes from Atlantic ones gives further weight to the assumption that the Orinoco River may act as a phylogeographic barrier to *M. rapax*, as proposed for other organisms [71, 72]. An increased sample size of both Venezuelan and Colombian animals would greatly help resolve this matter.

Levels of differentiation are high between the two geographic regions (here defined as Atlantic vs. Caribbean) in both species (24.09%/45.14% variation among regions as calculated with AMOVA) (Tables 1 and 4, respectively), while there is little to no differentiation detected among Atlantic populations (*p* > 0.5 in both species). Owing to these heterogeneous results, no general conclusion can be drawn on the question if their extended PLD predestinates *M. rapax* and *L. leptodactyla* to manifest strong genetic connection, or if these species are commonly prone to phylogeographic barriers. Shanks and colleagues [73, 74], concluded that PLD is incontestably a crucial factor in the dispersal of planktonic larvae. Nonetheless, the effective dispersal potential of a species proves rather difficult, if not impossible, to anticipate when factoring solely its PLD, especially if it lasts longer than one week. Rather than being mere passive particles drifting on random ocean currents, larvae may actively vertically migrate in the water column [74–76]. This behavior allows larvae to influence being either retained or dispersed [77, 78]. Surface ocean currents are faster, favoring migration, while near-bottom layers tend to run slower, countervailing dispersal (see [74], and citations therein). Some larvae may even oscillate between water layers, which often flow in different directions, thus further retarding advection [79]. For example, offspring of *Callinectes sapidus* or *Scylla serrata* is retained in near-shore waters [26, 80], while *Carcinus maenas* larvae are exported [81].

### *Minuca* *rapax*

It remains contested which strategy *M. rapax* larvae adopt and if the same strategy is used throughout different geographic areas. Populations along the coastal Atlantic habitats proved genetically homogeneous, hence, panmixia seems unimpaired. Contrasting, Caribbean populations are rather heterogeneous with a phylogenetic break being present somewhere around the Orinoco River area. It may be that larvae are frequently exported along the mainland shores, ensuring utter genetic exchange among even widespread populations, while offspring is retained in natal areas within the Caribbean. Ecological differences might thereby play a non-negligible role. When drifting over large distances, the risk is elevated to be washed off to habitats unsuitable for metamorphosing into adult crabs [5]. A possible explanation could thus be that in the Caribbean, suitable habitats are rather discontinuous, while being more abundant along the western Atlantic coastline. The restricted but extant gene flow among Caribbean populations could be owing to few individuals being exported, with the vast majority of larvae being retained within parental habitat [74]. Alternatively, assuming all offspring to emmigrate, only few larvae may actually be transported to suitable habitats, whereas all others may arrive in hostile surroundings (see [5] and citations therein).

Recent studies (e.g. [39]) revealed genetic homogeneity in other fiddler crabs along the southwestern Atlantic coast. Nonetheless, significant morphometric variance was detected. Similarly, phenotypic variation among here studied *M. rapax* populations was observed (e.g. some Jamaican individuals were much larger and had different coloration), although not yet statistically analyzed. Thus, statistical analyses on morphology are highly encouraged, including both morphometrics and trophic morphology to elucidate possible ecological adaptations and/or phenotypic plasticity as for example found in cave living crabs [82], as well as in other fiddler crabs [39, 40, 47]. Habitat variations within a broad distributional range that are not sufficiently large when related to effective dispersal, more likely result in phenotypic plasticity rather than actual local adaptation [83]. Significant differences detectable in morphometrics but not in genetics may also indicate ecological speciation in progress [57].

### *Leptuca* *leptodactyla*

Similar to findings in *M. rapax* (see also [46]), a deep division becomes visible between Caribbean and Atlantic populations of *L. leptodactyla*. No haplotypes are shared between these regions and pairwise differences indicate substantial restriction of genetic connectivity (*ϕ*_*ST*_ values approx. 0.5, Table 4). Two individuals from Venezuela each carry a distinct rare haplotype that find their closest relative (more than 20 mutations) within the Atlantic cluster. Misidentification can be excluded as no other Western Atlantic fiddler crab has similar cytochrome oxidase sequences (Laurenzano & Schubart, unpubl. data). The heterogeneity of the Brazilian populations with almost no shared haplotypes explains why variation is highest within populations, while second highest among regions (Brazil vs. Caribbean), whereas variation among population within regions is extremely low (52.68% variation within populations, 45.14% variation among regions, while only 2.18% variation among populations within regions, as calculated with AMOVA, Table 4). No differentiation was detected among Caribbean populations (Table 3).

The comparison between regions highly supports one of the biogeographic boundaries suggested by Briggs [63]. Whether or not the discharge of Amazon and Orinoco rivers play a major role in shaping a barrier between these distinct regions, as suggested, remains contested. Nonetheless, our results give further indication that these particular hydrographic phenomena may form an obstacle to larval exchange. The immense outflow carries freshwater up to 500 km seaward [84], possibly washing migrating larvae far offshore. Other physical aspects of the plume, such as altered temperature and salinity may also be deleterious to larval survival [10]. Within the area between the two rivers, i.e. French- Guyana, Suriname, and Guyana, *L. leptodactyla* shows a clear gap in distribution [41, 42, 45, 85], further corroborating the existence of a boundary. Long-term divergence as in species pairs from the respective sides of these rivers (i.e. Caribbean and Brazilian counterparts) is found in several faunal groups [86–91], leading [22] to attribute a great part of the encountered endemism in Brazil’s coastal fauna to the Amazon River freshwater plume.

Gene flow restriction between Caribbean and Atlantic *L. leptodactyla* seems absolute (no haplotype sharing, high *ϕ*_*ST*_ values), but not very old, as indicated by the short distances in the haplotype network. Hence, the zoogeographic barrier jointly constituted by the Amazon and Orinoco rivers may be intermittent, as suggested by [22]. During the interchange of glacial and interglacial periods, sea levels and subsequently salinity alter. This way, larval exchange between the Caribbean and Brazil may be strongly impaired, if not impossible, during ice ages, this way favoring differentiation of populations on the respective sides. With higher sea level, however, transgression may be facilitated, permitting gene flow between the two distinct regions. [92] detected communities of sponges and deep-water reef fishes in the deep outer shelf of the Amazon plume during high sea level, whose settlement most likely was enabled by high sedimentation and low sea level salinity. Light conditions are deficient for coral growth, thus, this can function as passage for northward migration at high sea levels [22]. This phenomenon may offer a possible explanation for the distribution pattern of *L. leptodactyla*, as well as the apparent population structuring observed between Atlantic and Caribbean populations.

Fiddler crab larvae may have the potential to cross the Amazon-Orinoco region, possibly enhanced by the strong flow of the North Brazilian Current, as proposed for Brazilian reef fishes [13]. [22] also suggests that speciation took place in the South Atlantic region followed by colonization of the Caribbean after passing the river plume, as many species are highly abundant in the Brazilian Province, while less widespread within the Caribbean or West Indian provinces [93]. This theory may also hold true for *L. leptodactyla* which is found throughout the tropical region southeast of the Amazon and great parts of the Caribbean Sea [41], but has not been reported from most of the Lesser Antilles. Contemporary sea levels were reached approximately 6,000 ya, while global temperatures started rising around 18,000 ya after the Wisconsin glacial epoch of roughly 100,000 years [94]. Molecular clock estimates would be helpful to determine if the division between Caribbean and Brazilian populations was chronologically correlated with the Wisconsin or preceding glacials, and should be considered for future studies. Assuming a temporally rather novel permeability of the barrier in a northward direction, *L. leptodactyla* populations that found a glacial refugium in Brazilian coastal habitats should exhibit a genetic structure much alike the one presented in this study.

### Conclusions

Our data suggest that gene flow is not entirely unimpaired among populations of *M. rapax* and *L. leptodactyla*. Both species show significant restrictions in genetic exchange between Caribbean and Atlantic populations which may indicate that the Orinoco, possibly enhanced by the Amazon, may function as a biogeographic barrier to dispersal. The Amazon alone, however, seems not to impede larval exchange, as both species exhibit ongoing gene flow. Within the Caribbean, contrasting patterns become obvious. While there is no evidence for genetic structuring in *L. leptodactyla* in this region, the opposite is true for *M. rapax*. Not only did we find significantly restricted gene flow among populations in this region, but a severe lack of genetic interchange between Hispaniola and Puerto Rico seems to be the case. This supports the suggestion that the Mona Passage may indeed function as barrier for this fiddler crab.

## References

[1] Epifanio CE. Transport of crab larvae between estuaries and the continental shelf In: Coastal-Offshore Ecosystem Interactions. Washington, D.C.: American Geophysical Union; 1988 p. 291–305. 10.1029/LN022p0291

[2] Epifanio CE, Little KT, Rowe PM. Dispersal and recruitment of fiddler crab larvae in the Delaware River estuary. Mar Ecol-Prog Ser. 1988;43:181–188. 10.3354/meps043181

[3] Palumbi SR. Macrospatial genetic structure and speciation in marine taxa with high dispersal abilities In: Ferris JD, Palumbi SR, editors. Molecular zoology: advances, strategies, and protocols. Wiley-Liss; 1996 p. 101–113.

[4] Avise JC. Molecular Markers: Natural history and evolution. Sunderland, Massachusetts: Sinauer; 1994 10.1007/978-1-4615-2381-9

[5] Palumbi SR. Genetic divergence, reproductive isolation, and marine speciation. Annu Rev Ecol Syst. 1994;25:547–572. 10.1146/annurev.es.25.110194.002555

[6] Scheltema RS. Larval dispersal as a means of genetic exchange between geographically separated populations of shallow-water benthic marine gastropods. Biol Bull. 1971;140:284–322. 10.2307/1540075

[7] Berger EM. Gene-enzyme variation in three sympatric species of *Littorina*. Biol Bull. 1973;145:83–90.

[8] Gooch JL. Mechanisms of evolution and population genetics In: Kinne O, editor. Marine ecology: a comprehensive, integrated treatise on life in oceans and coastal waters: 2. Wiley, London; 1975 p. 349–409.

[9] Crisp JD. Genetic consequences of different reproductive strategies in marine invertebrates In: Battaglia B, Beardmore J, editors. Marine organisms: genetics, ecology and evolution. New York: Plenum Press; 1978 p. 257–273.

[10] Hedgecock D. Is gene flow from pelagic larval dispersal important in the adaptation and evolution of marine invertebrates? B Mar Sci. 1986;39(2):550–564.

[11] Kyle CJ, Boulding EG. Comparative population genetic structure of marine gastropods (*Littorina* spp.) with and without pelagic larval dispersal. Mar Biol. 2000;137(5-6):835–845. 10.1007/s002270000412

[12] Taylor MS, Hellberg ME. Genetic evidence for local retention of pelagic larvae in a Caribbean reef fish. Science. 2003;299(5603):107–109. 10.1126/science.1079365 12511651

[13] Rocha LA, Robertson DR, Roman J, Bowen BW. Ecological speciation in tropical reef fishes. P R Soc B. 2005;272(1563):573–579. 10.1098/2004.3005 15817431 PMC1564072

[14] Baums IB, Paris CB, Chérubin LM. A bio-oceanographic filter to larval dispersal in a reef-building coral. Limnol Oceanogr. 2006;51(5):1969–1981. 10.4319/lo.2006.51.5.1969

[15] Weersing K, Toonen RJ. Population genetics, larval dispersal, and connectivity in marine systems. Mar Ecol-Prog Ser. 2009;393:1–12. 10.3354/meps08287

[16] Gaylord B, Gaines SD. Temperature or Transport? Range limits in marine species mediated Solely by Flow. Am Nat. 2000;155(6):769–789. 10.1086/303357 10805643

[17] Poulin E, Palma AT, Leiva G, Narvaez D, Pacheco R, Navarrette SA, et al Avoiding offshore transport of competent larvae during upwelling events: The case of the gastropod *Concholepas concholepas* in central Chile. Limnol Oceanogr. 2002;47(4):1248–1255.

[18] Taylor MS, Hellberg ME. Comparative phylogeography in a genus of coral reef fishes: biogeographic and genetic concordance in the Caribbean. Mol Ecol. 2006;15(3):695–707. 10.1111/j.1365-294X.2006.02820.x 16499695

[19] Colin PL. The neon gobies: the comparative biology of the gobies of the genus *Gobiosoma*, subgenus *Elacitunus*, (Pisces: Gobiidae) in the tropical North Atlantic Ocean. TFH Publications 1975

[20] Starck II, Walter A, Colin PL. *Gramma linki*: a new species of grammid fish from the tropical western Atlantic. B Mar Sci. 1978;28(1):146–152.

[21] Briggs JC. Marine Zoogeography. New York: McGraw-Hill; 1974.

[22] Rocha LA. Patterns of distribution and processes of speciation in Brazilian reef fishes. J Biogeogr. 2003;30(8):1161–1171. 10.1046/j.1365-2699.2003.00900.x

[23] Terossi M, Mantelatto FL. Morphological and genetic variability in Hippolyte obliquimanus Dana, 1852 (Decapoda, Caridea, Hippolytidae) from Brazil and the Caribbean Sea. Crustaceana. 2012;85(6):685–712. 10.1163/156854012X643762

[24] Shih HT, Ng PKL, Davie PJF, Schubart CD, Türkay M, Naderloo R, Jones D., Liu M. Systematics of the family Ocypodidae Rafinesque, 1815 (Crustacea: Brachyura), based on phylogenetic relationships, with a reorganization of subfamily rankings and a review of the taxonomic status of *Uca* Leach, 1814, sensu lato and its subgenera. Raffles B Zool. 2016;64:139–175.

[25] Morgan SG. Adaptive significance of hatching rhythms and dispersal patterns of estuarine crab larvae: avoidance of physiological stress by larval export? J Exp Mar Biol Ecol. 1987;113:71–78.

[26] Morgan SG, Christy JH. Adaptive significance of the timing of larval release by crabs. Am Nat. 1995;145(3):457–479. 10.1086/285749

[27] Christy JH, Morgan SG. Estuarine immigration by crab postlarvae: mechanisms, reliability and adaptive significance. Mar Ecol-Prog Ser. 1998;174:51–65.

[28] Morgan SG, Anastasia JR. Behavioral tradeoff in estuarine larvae favors seaward migration over minimizing visibility to predators. Proc Natl Acad Sci USA. 2008;105(1):222–227. 10.1073/pnas.0704725105 18172217 PMC2224190

[29] Hyman OW. The development of *Gelasimus* after hatching. J Morphol. 1920;33(2):1–42. 10.1002/jmor.1050330207

[30] Williams AB. Shrimps, lobsters, and crabs of the Atlantic coast of the eastern United States, Maine to Florida. Washington, D.C.: Smithsonian Institution Press; 1984.

[31] Christy JH. Adaptive significance of semilunar cycles of larval release in fiddler crabs (genus *Uca*): test of an hypothesis. Biol Bull. 1982;163:251–263.

[32] Strathmann RR. Selection for retention or export of larvae in estuaries In: Kennedy VS, editor. Estuarine Comparisons. Academic Press, New York; 1982 p. 521–536. 10.1016/B978-0-12-404070-0.50037-5

[33] Behum ME, Brodie RJ, Staton JL. Distribution of juvenile *Uca pugnax* and *U. pugilator* across habitats in a South Carolina estuary, assessed by molecular techniques. Mar Ecol-Prog Ser. 2005;288:211–220. 10.3354/meps288211

[34] Brodie RJ, Behum ME, Monroe E, Glenn N, Staton JL. Recruitment to adult habitats following marine planktonic development in the fiddler crabs, *Uca pugilator*, *U*. *pugnax* and *U. minax*. Mar Biol. 2005;147(1):105–111. 10.1007/s00227-005-1557-1

[35] Silva IC, Mesquita N, Paula J. Lack of population structure in the fiddler crab *Uca annulipes* along an East African latitudinal gradient: genetic and morphometric evidence. Mar Biol. 2010;157(5):1113–1126. 10.1007/s00227-010-1393-9

[36] Laurenzano C, Farias NE, Schubart CD. Mitochondrial genetic structure of two populations of *Uca uruguayensis* fails to reveal an impact of the Rio de la Plata on gene flow. Nauplius. 2012;20(1):15–25.

[37] Spivak ED. Los crustáceos decápodos del Atlántico sudoccidental (25°-55°S): distribución y ciclos de vida. Invest Mar. 1997;25:69–91.

[38] Boschi EE. Species of Decapod Crustaceans and their distribution in the American marine zoogeographic provinces. Rev Invest Desarr Pesq. 2000;13.

[39] Wieman AC, Berendzen PB, Hampton KR, Jang J, Hopkins MJ, Jurgenson J, et al A panmictic fiddler crab from the coast of Brazil? Impact of divergent ocean currents and larval dispersal potential on genetic and morphological variation in *Uca maracoani*. Mar Biol. 2013;161(1):173–185. 10.1007/s00227-013-2327-0

[40] Hampton KR, Hopkins MJ, McNamara JC, Thurman CL. Intraspecific variation in carapace morphology among fiddler crabs (Genus *Uca*) from the Atlantic coast of Brazil. Aquat Biol. 2014;20:53–67. 10.3354/ab00545

[41] Crane J. Fiddler crabs of the world: Ocypodidae: genus *Uca*. Princeton: Princeton University Press; 1975 10.1515/9781400867936

[42] Barnwell FH, Thurman CL. Taxonomy and biogeography of the fiddler crabs (Ocypodidae: Genus *Uca*) of the Atlantic and Gulf coasts of eastern North America. Zool J Linn Soc. 1984;81(1):23–87. 10.1111/j.1096-3642.1984.tb02558.x

[43] Salmon M, Kettler MK. The importance of behavioral and biochemical differences between fiddler crab taxa, with special refence to *Uca rapax* (Smith) and *U. virens* (Salmon and Atsaides). Contrib Mar Sci. 1987;30:63–76.

[44] Thurman CL. Fiddler crabs (genus *Uca*) of eastern Mexico (Decapoda, Brachyura, Ocypodidae). Crustaceana. 1987;53(1). 10.1163/156854087X00664

[45] Thurman CL, Faria SC, McNamara JC. The distribution of fiddler crabs (*Uca*) along the coast of Brazil: implications for biogeography of the western Atlantic Ocean. Mar Biodivers Rec. 2013;6:e1 10.1017/S1755267212000942

[46] Laurenzano C, Mantelatto FLM, Schubart CD. South American homogeneity versus Caribbean heterogeneity: population genetic structure of the western Atlantic fiddler crab *Uca rapax* (Brachyura, Ocypodidae). J Exp Mar Biol Ecol. 2013;449:22–27. 10.1016/j.jembe.2013.08.007

[47] Hopkins MJ, Thurman CL. The geographic structure of morphological variation in eight species of fiddler crabs (Ocypodidae: genus *Uca*) from the eastern United States and Mexico. Biol J Linn Soc. 2010;100:248–270. 10.1111/j.1095-8312.2010.01402.x

[48] de Jesus de Brito Simith D, Pires MAB, Abrunhosa FA, Maciel CR, Diele K. Is larval dispersal a necessity for decapod crabs from the Amazon mangroves? Response of Uca rapax zoeae to different salinities and comparison with sympatric species. J Exp Mar Biol Ecol. 2014;457(C):22–30.

[49] Schubart CD. Mitochondrial DNA and Decapod Phylogenies: The Importance of Pseudogenes and Primer Optimization In: Martin JW, Crandall KA, Felder DL, editors. Decapod Crustacean Phylogenetics. CRC Press Llc.; 2009 p. 47–65. 10.1201/9781420092592-c4

[50] Hall TA. BioEdit: a user-friendly biological sequence alignment editor and analysis program for Windows 95/98/NT. Nucl Acid S. 1999;41:95–98.

[51] Rutherford K, Parkhill J, Crook J, Horsnell T, Rice P, Rajandream MA, et al Artemis: sequence visualization and annotation. Bioinformatics. 2000;16(10):944–945. 10.1093/bioinformatics/16.10.944 11120685

[52] Clement M., Posada D., and Crandall K. A. (2000). TCS: a computer program to estimate gene genealogies. *Mol Ecol*, 9:1657–1659. 11050560 10.1046/j.1365-294x.2000.01020.x

[53] Excoffier L, Laval G, Schneider S. Arlequin (version 3.0): An integrated software for population genetics data analysis. Evol Bioinform. 2005;1:47–50. 19325852 PMC2658868

[54] Peterson RG, Stramma L. Upper-level circulation in the South Atlantic Ocean. Prog Oceanog. 1991;26:1–73. 10.1016/0079-6611(91)90006-8

[55] Barton N. Genetic revolutions, founder effects, and speciation. Annu Rev Ecol Syst. 1984;15:133–164. 10.1146/annurev.es.15.110184.001025

[56] Avise JC. Phylogeography The History and Formation of Species. Harvard University Press; 2000 10.1006/rwgn.2001.1470

[57] Galligan TH, Donnellan SC, Sulloway FJ, Fitch AJ, Bertozzi T, Kleindorfer S. Panmixia supports divergence with gene flow in Darwin’s small ground finch, *Geospiza fuliginosa*, on Santa Cruz, Galápagos Islands. Mol Ecol. 2012;21(9):2106–2115. 10.1111/j.1365-294X.2012.05511.x 22404597

[58] Gusmão J, Lazoski C, Solé-Cava AM. Population genetic structure of Brazilian shrimp species (*Farfantepenaeus sp*., *F*. *brasiliensis*, *F*. *paulensis and Litopenaeus schmitti*: Decapoda: Penaeidae). Genet Mol Biol. 2005;28(1):165–171.

[59] Ituarte RB, D’Anatro A, Luppi TA, Ribeiro PD, Spivak ED, Iribarne OO, et al Population Structure of the SW Atlantic Estuarine Crab *Neohelice granulata* Throughout Its Range: a Genetic and Morphometric Study. Estuar Coast. 2012;35(5):1249–1260. 10.1007/s12237-012-9516-9

[60] Mitton JB, Berg CJ Jr, Orr KS. Population Structure, Larval Dispersal, and Gene Flow in the Queen Conch, *Strombus gigas*, of the Caribbean. Biol Bull. 1989;177:356–362. 10.2307/1541595

[61] Silberman JD, Sarver SK, Walsh PJ. Mitochondrial DNA variation and population structure in the spiny lobster *Panulirus argus*. Mar Biol. 1994;120(4):601–608. 10.1007/BF00350081

[62] Shulman MJ, Bermingham E. Early Life Histories, Ocean Currents, and the Population Genetics of Caribbean Reef Fishes. Evolution. 1995;49(5):897–910. 10.2307/2410412 28564869

[63] Briggs JC. Global Biogeography Developments in Paleontology and Stratigraphy. Amsterdam: Elsevier; 1995.

[64] Galetti PM, Molina WF, Affonso PRAM, Aguilar CT. Assessing genetic diversity of Brazilian reef fishes by chromosomal and DNA markers. Genetica. 2006;126(1-2):161–177. 10.1007/s10709-005-1446-z 16502093

[65] Cowen RK, Paris CB, Srinivasan A. Scaling of Connectivity in Marine Populations. Science. 2006;311:522–527. 10.1126/science.1122039 16357224

[66] Díaz-Ferguson E, Haney R, Wares J, Silliman B. Population Genetics of a Trochid Gastropod Broadens Picture of Caribbean Sea Connectivity. PLoS ONE. 2010;5(9):e12675 10.1371/journal.pone.0012675 20844767 PMC2937038

[67] Brazeau DA, Lesser MP, Slattery M. Genetic structure in the coral, *Montastraea cavernosa*: assessing genetic differentiation among and within Mesophotic reefs. PLoS ONE. 2013;8(5):e65845 10.1371/journal.pone.0065845 23734263 PMC3666989

[68] Colin PL. Larvae retention: genes or oceanography? Science. 2003;300 10.1126/science.300.5626.1657c 12805519

[69] Dennis GD, Smith-Vaniz WF, Colin PL, Hensley DA, McGehee MA. Shore fishes from islands of the Mona Passage, Greater Antilles with comments on their zoogeography. Caribb J Sci. 2005;41(4):716–743.

[70] Baums IB, Miller MW, Hellberg ME.Regionally isolated populations of an imperiled Caribbean coral, *Acropora palmata*. Mol Ecol. 2005;14(5):1377–1390. 10.1111/j.1365-294X.2005.02489.x 15813778

[71] Gilbert CR. Characteristics of the western Atlantic reef-fish fauna. Quarterly Journal of Florida Academy of Sciences. 1972;35:130–144.

[72] Lessios HA, Kane J, Robertson DR. Phylogeography of the pantropical sea urchin *Tripneustes*: Contrasting patterns of population structure between oceans. Evolution. 2003;57(9):2026–2036. 10.1554/02-681 14575324

[73] Shanks AL, Grantham BA, Carr MH. Propagule dispersal distance and the size and spacing of marine reserves. Ecol Appl. 2003;13(1):159–169. 10.1890/1051-0761(2003)013[0159:PDDATS]2.0.CO;2

[74] Shanks AL. Pelagic larval duration and dispersal distance revisited. Biol Bull. 2009;216(3):373–385. 10.1086/BBLv216n3p373 19556601

[75] Koehn RK. Esterase Heterogeneity: Dynamics of a Polymorphism. Science. 1969;163:943–944. 10.1126/science.163.3870.943 5763877

[76] McMillen-Jackson AL, Bert TM, Steele P. Population genetics of the blue crab *Callinectes sapidus*: modest population structuring in a background of high gene flow. Mar Biol. 1994;118:53–65.

[77] Warner RR, Cowen RK. Local retention of production in marine populations: evidence, mechanisms, and consequences. B Mar Sci. 2002;70(1):245–249.

[78] Palumbi SR. Population genetics, demographic connectivity, and the design of marine reserves. Ecol Appl. 2003;13(1):146–158. 10.1890/1051-0761(2003)013[0146:PGDCAT]2.0.CO;2

[79] Largier JL. Considerations in estimating larval dispersal distances from oceanographic data. Ecol Appl. 2003;13(1):71–89. 10.1890/1051-0761(2003)013[0071:CIELDD]2.0.CO;2

[80] Webley JAC, Connolly RM. Vertical movement of mud crab megalopae (*Scylla serrata*) in response to light: Doing it differently down under. J Exp Mar Biol Ecol. 2007;341(2):196–203. 10.1016/j.jembe.2006.10.001

[81] Queiroga H, Blanton J. Interactions Between Behaviour and Physical Forcing in the Control of Horizontal Transport of Decapod Crustacean Larvae. Adv Mar Biol. 2004;47:107–214. 10.1016/S0065-2881(04)47002-3 15596167

[82] Stemmer M, Schubart CD. Allopatric differentiation and morphometric growth in a Jamaican freshwater crab, with the discrimination of a cave phenotype. Stud Neotrop Fauna E. 2013;48(2):95–103. 10.1080/01650521.2013.835614

[83] Sotka EE. Natural selection, larval dispersal, and the geography of phenotype in the sea. Integr Comp Biol. 2012;52(4):538–545. 10.1093/icb/ics084 22634357

[84] Lentz SJ. The Amazon River Plume during AMASSEDS: Subtidal current variability and the importance of wind forcing. J Geophys Res. 1995;100(C2):2377 10.1029/94JC00343

[85] Powers LW. Crabs (Brachyura) of the Gulf of Mexico. Contrib Mar Sci. 1977;20:1–189.

[86] Emery AR. Atlantic bicolor damselfish (Pomacentridae): a taxonomic question. Copeia. 1973;1973(3):590–594. 10.2307/1443127

[87] Gilbert CR. Status of the Western South Atlantic Apogonid Fish *Apogon americanus*, with Remarks on Other Brazilian Apogonidae. Copeia. 1977;1977(1):25–32. 10.2307/1443500

[88] Sarver SK, Silberman JD, Walsh PJ. Mitochondrial DNA sequence evidence supporting the recognition of two subspecies or species of the Florida spiny lobster *Panulirus argus*. Journal of Crustacean Biology. 1998;18(1):177–186. 10.2307/1549532

[89] Muss A, Robertson DR, Stepien CA, Wirtz P, Bowen BW. Phylogeography of *Ophioblennius*: the role of ocean currents and geography in reef fish evolution. Evolution. 2001;55(3):561–572. 10.1554/0014-3820(2001)055[0561:POOTRO]2.0.CO;2 11327163

[90] Rocha LA, Guimarães R, Gasparini JL. Redescription of the brazilian wrasse *Thalassoma noronhanum* (Boulenger, 1890) (Teleostei: Labridae). Aqua. 2001;4(3):105–108.

[91] Rocha LA, Bass AL, Robertson DR, Bowen BW. Adult habitat preferences, larval dispersal, and the comparative phylogeography of three Atlantic surgeonfishes (Teleostei: Acanthuridae). Mol Ecol. 2002;11(2):243–252. 10.1046/j.0962-1083.2001.01431.x 11856425

[92] Collette BB, Rützler K. Reef fishes over sponge bottoms off the mouth of the Amazon River. In: Proceedings of the Third International Coral Reef Symposium; 1977.

[93] Rocha LA. Brazilian reef fishes In: Humann P, Deloach N, editors. Reef Fish Identification. New World Publications; 2002 p. 462–479.

[94] Cox CB, Moore PD. Biogeography. An ecological and evolutionary approach. John Wiley & Sons; 2000.

